# Cervical mixed small cell neuroendocrine carcinoma and adenocarcinoma with genital tract dissemination: a case report and literature review

**DOI:** 10.3389/fonc.2026.1911426

**Published:** 2026-08-12

**Authors:** Ping Liu, Hong Fang, Xiaolin Tang, Qingke Dong, Taohong Xie, Junjian Feng

**Affiliations:** 1 Department of Pathology, Luzhou People’s Hospital, Luzhou, Sichuan, China; 2 Department of Hematology, Luzhou People’s Hospital, Luzhou, Sichuan, China; 3 Department of Gynecology, Luzhou People’s Hospital, Luzhou, Sichuan, China; 4 Department of Intensive Care Unit, Luzhou People’s Hospital, Luzhou, Sichuan, China

**Keywords:** adenocarcinoma, cervical neoplasms, human papillomavirus, mixed neuroendocrine-non-neuroendocrine neoplasms, small cell neuroendocrine carcinoma

## Abstract

**Background:**

Primary mixed neuroendocrine-non-neuroendocrine neoplasms (MiNENs) of the cervix are exceedingly rare. This report describes a case of human papillomavirus (HPV)-associated cervical MiNEN composed of adenocarcinoma and small cell neuroendocrine carcinoma (SCNEC) of cervix. Clinical and pathological findings suggest that the adenocarcinoma component extended to the endometrium, bilateral fallopian tubes, and ovaries via genital tract dissemination.

**Case presentation:**

A 48-year-old female presented with abnormal vaginal discharge for more than eight months and HPV genotyping test was positive for type 18. Pelvic MRI revealed cervical space-occupying lesions and cystic changes in the adnexal region. Gross examination revealed an ulcerative cervical mass, thickened and dilated fallopian tubes, and polycystic ovaries. The tumor was solid, and the cut surface was grayish white with mucus. Histologically, the cervical tumor comprised 65% SCNEC and 35% HPV-associated adenocarcinoma, both of which diffusely expressed p16. Adenocarcinoma was identified in the endometrium and bilateral fallopian tubes and ovaries, and its morphology, immunophenotype, and HPV infection status were consistent with those of cervical adenocarcinoma, suggesting that the cervical adenocarcinoma disseminated along the reproductive tract. Despite receiving systemic chemotherapy and local radiotherapy following surgery, the patient unfortunately passed away due to the progression of the disease in November 2022.

**Conclusions:**

Cervical MiNENs are highly aggressive malignancies associated with poor prognoses. HPV-associated tumors may spread contiguously through the reproductive tract, mimicking primary tumors of the endometrium, fallopian tubes, or ovaries. Accurate diagnosis and effective management of patients rely on meticulous postoperative histopathological evaluation.

## Background

Neuroendocrine neoplasms predominantly arise in the digestive tract and lungs and are rarely encountered in the cervix, constituting approximately 0.5% to 1% of all cervical malignancies (1–5). According to the 2020 World Health Organization (WHO) Classification of Female Genital Tumors, neuroendocrine neoplasms of the cervix are categorized into low to intermediate-grade neuroendocrine tumors (NETs), which divided into Grade 1 and Grade 2 tumors, and high-grade neuroendocrine carcinomas (NECs), which include SCNEC and large cell neuroendocrine carcinoma (LCNEC). Additionally, MiNENs are recognized (6). According to the WHO, MiNENs comprise two histologically and immunophenotypically distinct components, one neuroendocrine and one non-neuroendocrine, each constituting at least 30% of the tumor mass. Cervical MiNENs have been reported only in individual cases and are typically composed of a combination of high-grade NECs and adenocarcinoma (7–28). This report presents a case of HPV18-associated adenocarcinoma mixed with SCNEC of the cervix. The adenocarcinoma had extended to the bilateral fallopian tubes and ovaries via the endometrium. We discuss its clinicopathological features in conjunction with the relevant literature to enhance the understanding of clinicians and pathologists.

## Case presentation

### Clinical history

**Figure 1:**
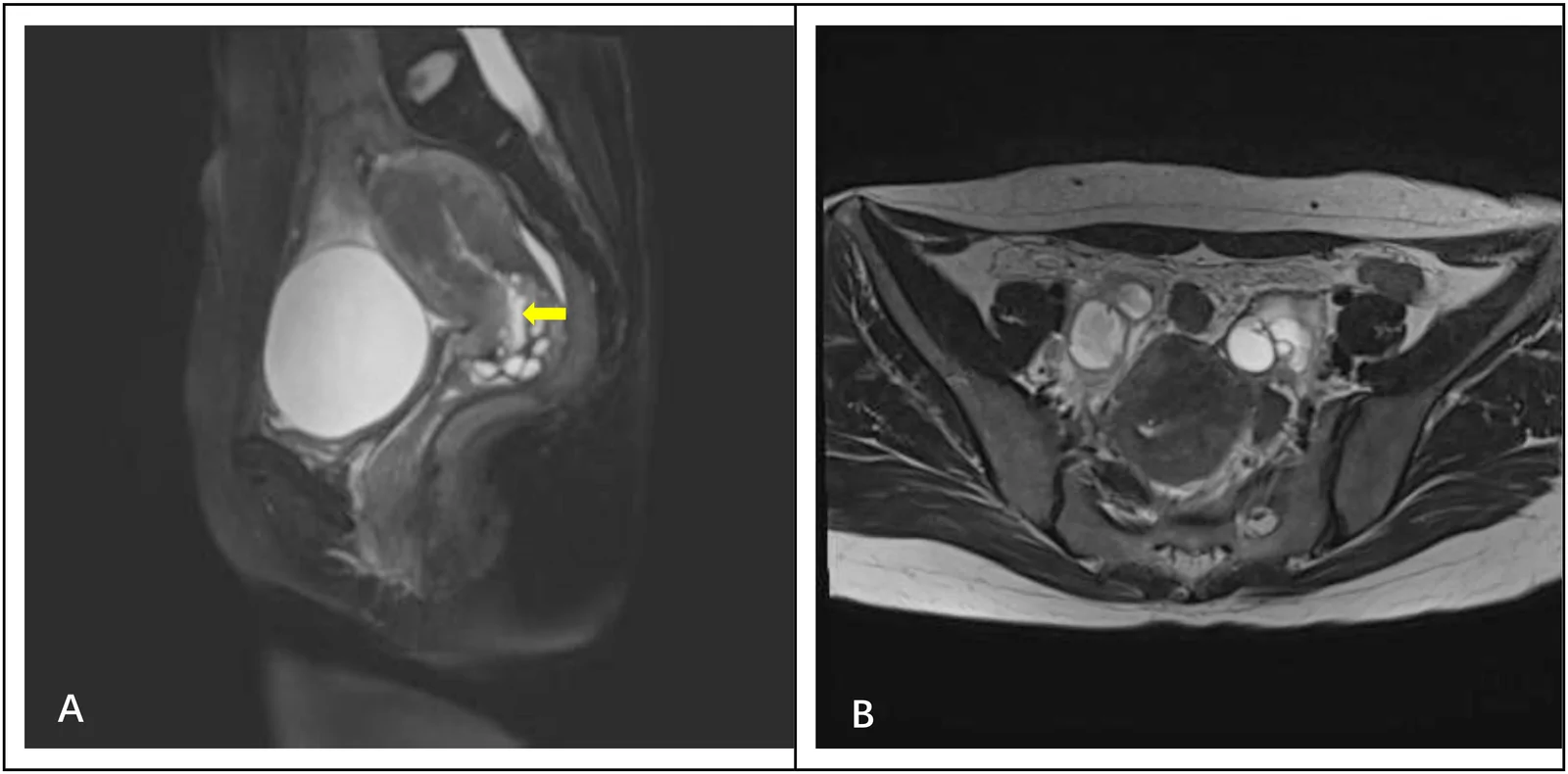
Imaging findings. **(A)** Pelvic MRI T2WI suggested a soft tissue mass and multiple small cysts of the cervix (yellow arrow). **(B)** Both ovaries were multilocular cystic with high signal on T2WI.

A 48-year-old female patient experienced significant vaginal discharge since January 2020, characterized by transparent and thin fluid without foul odor. Cervical cytology revealed no abnormalities, but HPV genotyping test was positive for type 18. Physical examination revealed a large amount of transparent mucus in the vagina. The cervix was barrel-thickened with a diameter of 4 cm. A neoplasm was identified in the cervical canal, which bled easily upon contact. Tumor marker levels were elevated: CA-125 (153.8 U/mL, normal <35), CA 19-9 (68.43 U/mL, normal <37), and ferritin (493.02 ng/mL, normal range varies by age and sex). Pelvic MRI (T2-weighted imaging) demonstrated a heterogeneous soft tissue mass in the cervix with multiple small cystic areas. The cervical stromal ring was disrupted, indicating deep stromal invasion (**Figure 1A**). The bilateral adnexal regions presented multilocular cystic lesions, and the cystic wall presented high signal intensity on T2-weighted imaging (**Figure 1B**), with extensive adherence to the peritoneum and intestines. Pathological examination of the cervical biopsy revealed the presence of small cell carcinoma, with no other components. The patient subsequently underwent radical hysterectomy, bilateral salpingo-oophorectomy, and pelvic lymph node dissection.

### Gross features

**Figure 2:**
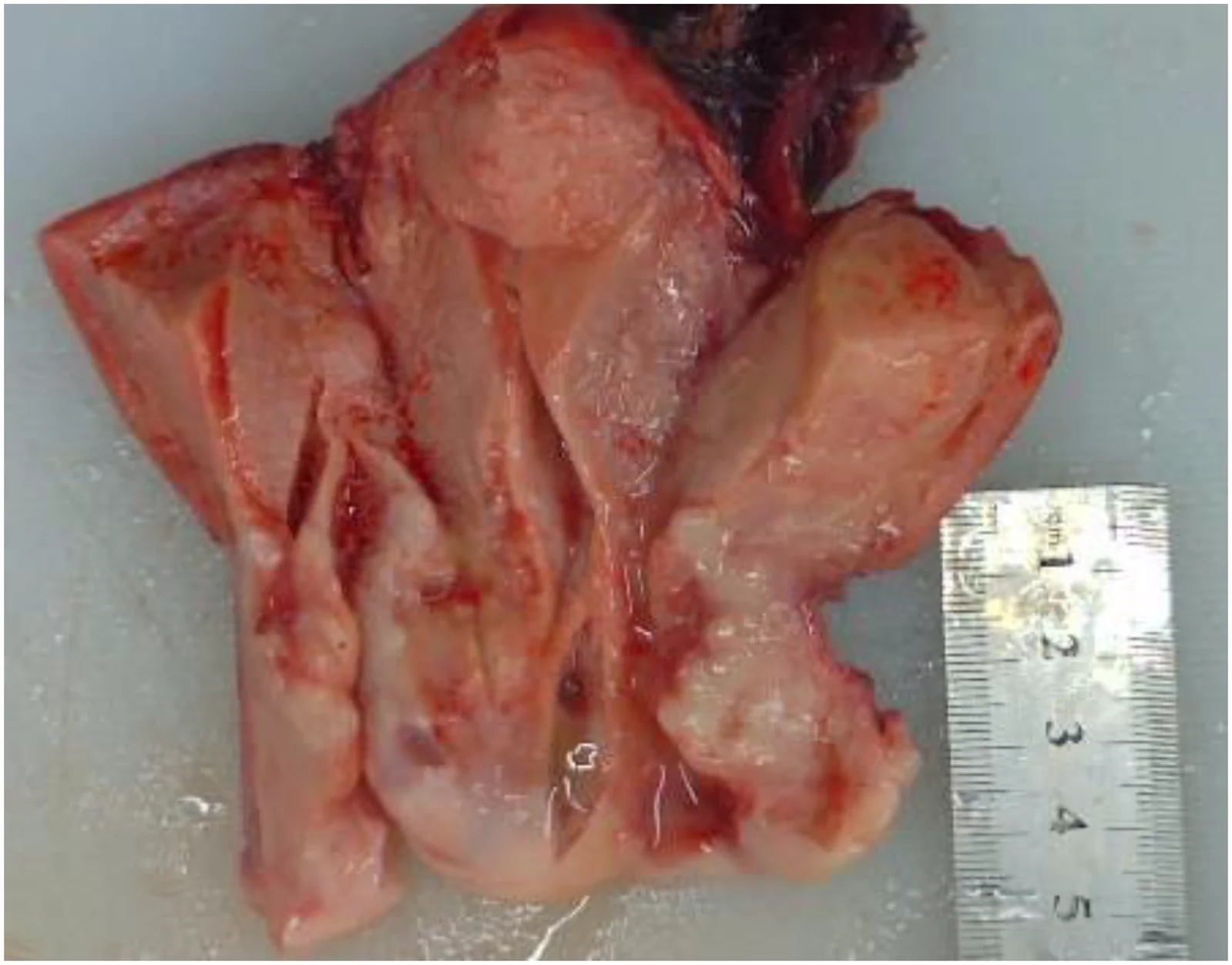
Grossly structures. The tumor was located in the cervical canal and invaded the deep cervical interstitial layer.

The specimen obtained from radical hysterectomy revealed a uterus measuring 5.7 cm × 5.5 cm × 3.8 cm, with an endometrial thickness of 0.1 cm. No macroscopic masses were seen in the endometrium and uterine myometrium. The cervix measured 4 cm in length by 3.8 cm in diameter and featured a smooth ectocervix. A 3.5 cm × 3 cm × 1.3 cm ulcerative mass was observed in the cervical canal, 0.6 cm from the external cervical os. The tumor had a gross depth of invasion of 1.3 cm, and histopathological examination confirmed that the tumor invaded the deep stroma of the cervix and extended to the lower uterine segment. The cut surface of the tumor appeared gray-white and solid and contained mucus (**Figure 2**). The bilateral fallopian tubes were dilated and twisted, with diameters ranging from 0.5 cm to 2 cm. The left ovary measured 4.2 cm × 2.7 cm × 2 cm, whereas the right ovary measured 9.5 cm × 8.2 cm × 3 cm. The cut surface of the ovary showed polycystic changes with smooth cyst walls and no vegetations. The cysts contain dark red mucoid fluid.

## Materials and methods

Surgical specimens were fixed in 10% buffered formalin, embedded in paraffin, and serially sectioned into 4-μm-thick slices. Routine hematoxylin and eosin (HE) staining was performed. Representative tissue blocks were also selected for immunohistochemical staining. All the antibodies were purchased from Fuzhou Maixin Company. The primary antibodies used included cytokeratin (CK), carcinoembryonic antigen (CEA), CD56, CDX-2, chromogranin A (CgA), CK5/6, estrogen receptor (ER), Ki-67, p16, p40, p53, progesterone receptor (PR), synaptophysin (Syn), thyroid transcription factor-1(TTF-1), vimentin and the wilm’s tumor gene (WT-1). HPV genotyping test was performed using the HPV Genotyping Detection Kit (PCR membrane hybridization method) manufactured by Guangdong Hybribio Biotech Co., Ltd., which can automatically identify 21 HPV subtypes. Representative paraffin tissue blocks of cervix, endometrium, fallopian tube and ovarian tumor tissues were selected. Three consecutive sections with 6-μm-thick were prepared and collected into centrifuge tubes for dewaxing and DNA extraction. Subsequent operations were carried out in accordance with the kit instructions, and the results were automatically analyzed and printed out by the instrument.

To systematically review previously reported cervical MiNEN cases, we searched the PubMed and Embase databases in December 2024 using the following keywords: ‘cervical’ AND ‘mixed neuroendocrine-non-neuroendocrine neoplasm’ OR ‘MiNEN’ OR ‘mixed neuroendocrine carcinoma.’ Inclusion criteria were: (1) histopathologically confirmed primary cervical MiNEN; (2) adequate clinicopathological data provided; (3) published in English. Exclusion criteria were: (1) incomplete case information; (2) duplicate publications; (3) non-cervical primary origin. A total of 26 cases meeting the criteria were identified and summarized in **Table 1**.

**Table 1:** Clinicopathological features of 27 cases of cervical MiNENs (7–28).

References	Age/ year	Tumor type	IHC		HPV	Treatment	FIGO	Metastasis	Follow-up
			Non-neuroendocrine	Neuroendocrine					
Toki (7)	42	SCNEC+MiSCC+AIS	CEA	NSE, CgA, Sertonin, CEA, Gastrin	–	RHT	IB1	※SCNEC metastasized to subcutaneous tissue of the abdomen	30 months DOD
Cetiner (8)	37	LCNEC+MAC+AIS	CEA, CK, EMA	NSE, CgA	/	RHT+ pelvic and para-aortic LND+ RT	IIA	※MAC metastasized to right pelvic lymph nodes	6 months NED
Alphandery (9)	41	SCNEC+AC	p16	p16, Syn, CD56, c-kit, CgA, Ki-67 80%	18	ChT +RHT	IIIA	/	/
Ko (10)	45	LCNEC+AC	/	Syn, NSE, CgA,	18	RHT+ pelvic and para-aortic LND+ ChT	IB2	No	24 months NED
Hao (11)	31	LCNEC+MAC (intestinal-type)	p16, CEA, MUC2, Ki-67 80%	p16, CgA, CD56, CEA, RB, TTF-1, Ki-67 80%	18	RHT+ ChT	IB1	No	/
Ramalingam (12)	38	SCNEC+AC	p16	p16, Syn	HR-HPV	RHT+ CRT	IVA	MAC metastasized to ovaries	59 months NED
	35	SCNEC+AC	p16	p16, Syn, CgA	HR-HPV	RHT+ ChT	IVA	MAC metastasized to ovaries, and SCNEC metastasized to liver	11 months NED
Yasuoka (13)	36	LCNEC+MAC (intestinal-type)	p16	p16, CgA	18	/	IB1	/	3 months NED
Nishimura (14)	36	SCNEC+AC	/	Syn, CgA, Ki-67 30%	/	RHT+ RT+ ChT	IB	※SCNEC metastasized to lung and pancreas	96 months NED
Fadıloğlu (15)	50	LCNEC+MAC	/	Syn, CgA, EMA, CK7, Ki-67 80%, PR(p+)	16	RHT+ pelvic and para-aortic LND+ ChT	IVB	LCNEC metastasized to ovaries	6 months NED
Cavalcanti (16)	59	SCNEC+MA	PAX-8, CD10, p16, GATA-3	p16, calretinin, Syn, CD56, PAX-8, CgA, GATA-3(p+)	–	RHT+ pelvic LND	IB1	No	14 months DOD
Kaushal (17)	36	SCNEC+SCC	p40	Syn, CD56, CgA, CK, Ki-67 100%	/	RHT + pelvic LND + ChT+ RT	IB3	No	12 months NED
Gill (18)	77	SCNEC+ACC	/	Syn	35	RHT + RT+ ChT+ IMT	IB2	※SCNEC metastasized to supraclavicular, paraaortic, pelvic lymphadenopathy and right hepatic	18 months DOD
Teoman (19)	56	SCNEC+AC	p16, CEA	p16, CEA, Syn, CD56, CgA	18	RHT	IIA	No	/
Ordulu (20)	32	LCNEC+MAC (intestinal-type)	Syn, CgA	Syn, CgA, CD56	16/18	RHT+ pelvic and para-aortic LND	IB2	/	/
	49	LCNEC+AC	/	Syn	16/18	RHT+ pelvic LND	IB1	/	58 months DOD
	66	LCNEC+AC	/	Syn, CgA	16/18	RHT+ pelvic and para-aortic LND	IIIC	No	12 months NED
XU (21)	57	LCNEC+AC	CK, CK8/18, p16	CK, CgA, Syn, CD56, p16, CEA, Ki-67 90%	18	RHT	IB2	/	/
Fattorini (22)	49	LCNEC+MAC (intestinal-type)	p16, CK7, CEA, CDX-2, PAX-8, Ki-67 50%	p16, Syn, ISNM1, hASH1, CDX2, TTF-1, CK7, CEA, Ki-67 90%	18	RHT + appendix and suspect liver nodule+ ChT+ RT	IVB	SCNEC metastasized to liver	13 months DOD
Gurubalan (23)	75	SCNEC+AC	p16, CK7	CK, NES, Syn		RHT + ChT	IB1	No	4 months NED
Wei (24)	54	LCNEC+GAS	CK, MUC6, PAX-8, HNF1β	CgA, Syn	–	RHT+ pelvic and para-aortic LND+ ChT+ RT	IIA1	AC metastasized to ovary and left sacral bone※	17 months NED
	50	LCNEC+AC	p16, CK, MLH1, MSH2, MSH6, PMS2, Ki-67 50-80%	p16, Syn, CD56, SSTR2, Ki-67 50-80%	18	ChT+ RHT+ pelvic and para-aortic LND+ ChT	IIIC1	LCNEC metastasized to pelvic lymph nodes	11 months NED
Molla (25)	40	SCNEC+AC	CK	CgA, Syn	/	ChT	IVB	AC metastasized to skin, lumbar vertebrae, and thyroid gland	/
Lamiman (26)	44	LCNEC+AC (usual-type)	p16	p16, CgA, Syn	+	RHT+ LND+ ChT+ RT	IB1	No	>96 months NED
Cai (27)	55	SCNEC+CEC +GAS	MUC6	Syn, CD56,	16, 18	RHT+ pelvic and para-aortic LND+ ChT+TKI	IB2	※CEC metastasized to lung	24 months NED
Alwaqfi (28)	33	NET G1+AC (intestinal-type)	p16, CDX-2, CK20, CK7, PAX-8	p16, Syn, CgA, INSM1, Ki-67 0%	16, 18	RHT	IA2	No	/
Present case	48	SCNEC+AC (usual-type)	p16, CK, CEA, Ki-67 65%	p16, Syn, CD56, CgA, CK, Ki-67 85%	18	RHT+ pelvic LND+ ChT+ RT	IB2	AC metastasized to ovaries	26 months DOD

AC, adenocarcinoma; ACC, adenoid cystic carcinoma; AIS, adenocarcinoma in situ; CEC, cervical endometrioid carcinoma; ChT, chemotherapy; DOD, dead of disease; GAS, gastric type adenocarcinoma; IMT, immunotherapy; LCNEC, large cell neuroendocrine carcinoma; LND, lymph node dissection; MA, mesonephric adenocarcinoma; MAC, mucinous adenocarcinoma; NED, no evidence of disease; NET G1, Grade 1 neuroendocrine tumor; RHT, radical hysterectomy; MiSCC, micro-invasive squamous cell carcinoma; SCNEC, small cell neuroendocrine carcinoma; TKI, tyrosine kinase inhibitor.

## Results

### Histologic features

**Figure 3:**
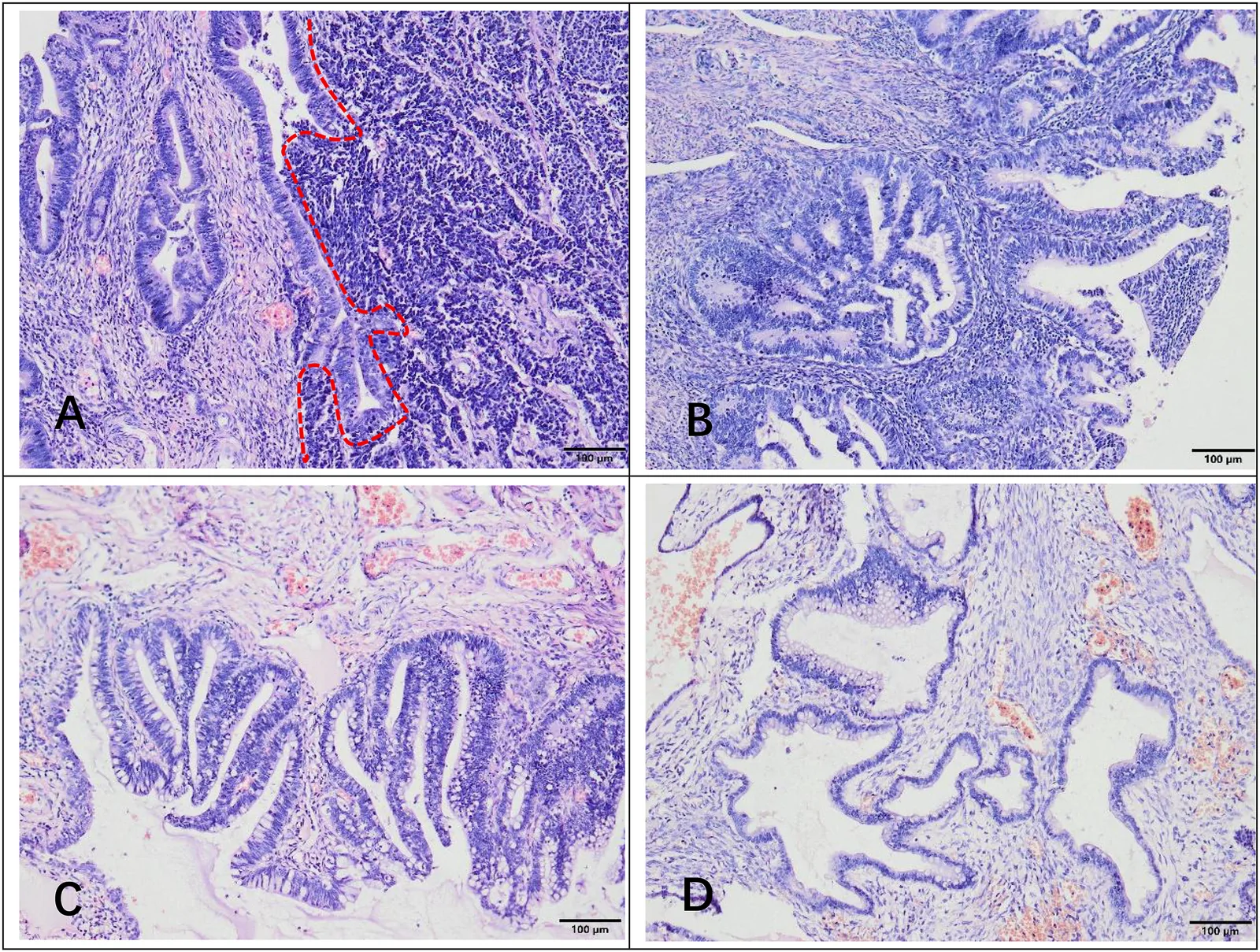
Morphological features of the tumor. **(A)** Adenocarcinoma (left of the red dotted line) and SCNEC (right of the red dotted line) coexisted within the same cervical tumor, with the two types of tumor cells closely adjacent. (100×). **(B)** Tumor of endometrial was confined solely to the endometrial layer (100×). **(C)** Tumor (right) proliferated along normal tubal mucosa (left), with marked morphological discrepancy (100×). **(D)** The glands of ovarian tumor were irregular. A higher proportion of mucous cells led to dilatation of glandular lumens, while mitotic figures could still be seen at the glandular lumen margins (100×).

Microscopically, cervical tumor was composed of 65% SCNEC and 35% adenocarcinoma, and the two types of tumor cells were closely adjacent and transitioning into each other (**Figure 3A**). SCNEC exhibited a diffuse growth pattern characterized by a high nuclear-to-cytoplasmic ratio and finely granular nuclear chromatin. Mitotic figures and necrosis were frequently observed. Intravascular tumor emboli and perineural invasion were exclusively associated with SCNEC. Adenocarcinoma displayed tubular, papillary, and cribriform patterns, with glandular ducts lined by columnar cells and a few mucinous cells. Mitotic figures and apoptotic bodies were noted at the apices of glandular structures, which conformed to the morphological characteristics of cervical HPV-associated adenocarcinoma. Only adenocarcinoma was identified as the histological type in the endometrium, fallopian tubes and ovaries, with no evidence of SCNEC. Morphologically, these tumors resembled primary adenocarcinomas arising from these sites. However, careful observation revealed consistent morphological features with cervical adenocarcinoma, and mitotic figures as well as apoptotic bodies were visible at the glandular luminal margins (**Figures 3B–D**). The tumors of endometrial and fallopian tube were confined to the mucosal layer, without invasion into the myometrium or blood vessels. Ovarian lesions were liable to be misdiagnosed as primary borderline mucinous tumors, owing to increased proportion of mucinous cells leading to glandular dilatation and formation of mucin pools. In addition, CIN III changes can be observed in the cervical squamous epithelium.

The morphological and immunophenotypic consistency between the adnexal and cervical adenocarcinoma strongly supports secondary involvement via transtubal or transepithelial spread rather than synchronous primary tumors.

### Immunohistochemical staining

**Figure 4:**
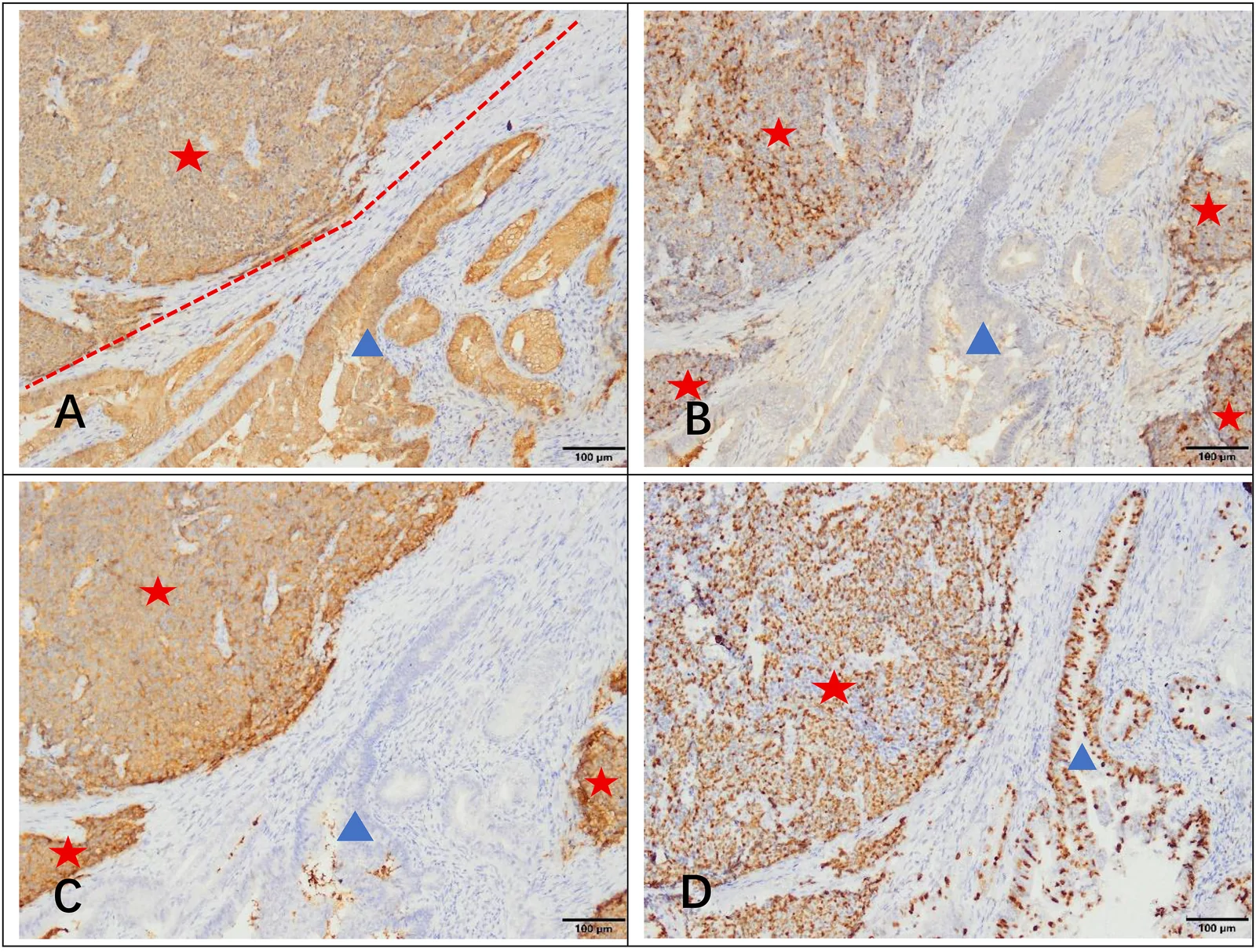
Immunohistochemical study of the two components. **(A)**, p16 exhibited diffuse and strong nuclear and cytoplasmic immunoreactivity in both the SCNEC (red five-pointed star, upper left of the red dotted line) and adenocarcinoma (blue triangle, lower right of the red dotted line) (100×). **(B)** CgA positivity was restricted exclusively to the SCNEC (red five-pointed star) component (100×). **(C)** Syn positivity was also restricted exclusively to the SCNEC (red five-pointed star) component (100×). **(D)** Both components exhibited a high proliferative index with Ki67 (100×).

Immunohistochemical staining showed that adenocarcinoma was diffusely positive for CK and p16 (**Figure 4A**), CEA was positive at glandular luminal margins. CDX-2, ER, PR, vimentin, and WT-1 were negative, p53 showed wild-type expression, and the tumor cell proliferation index Ki-67 was approximately 65%. SCNEC was positive for p16, CgA (**Figure 4B**), Syn (**Figure 4C**), and CD56. The CK staining pattern in the SCNEC was para-nuclear punctate positive, TTF-1 was weakly positive in some cells, CK5/6 and p40 were negative, and Ki-67 was present in approximately 80% of the cells (**Figure 4D**). HPV genotyping results indicated that HPV type 18 was detected in paraffin-embedded tissue samples from cervical, endometrial, fallopian tube and ovarian tumors, which strongly supports tumor dissemination along the genital tract.

### Pathological diagnosis

The pathological diagnosis was cervical mixed small cell neuroendocrine carcinoma-adenocarcinoma (MiNEN), with adenocarcinoma extending into the endometrium and both the fallopian tubes and ovaries. No metastatic disease was identified in the bilateral pelvic lymph nodes. The final FIGO stage was IB2.

### Follow-up

Postoperatively, the patient received radiotherapy targeting the vaginal stump and pelvic lymph node drainage area, along with six cycles of chemotherapy with etoposide (150mg/m²d1-3) and cisplatin (75mg/m²d1). Radiotherapy was delivered via intensity-modulated radiotherapy (IMRT) with a Swedish Elekta Precise linear accelerator employing 6-MV X-rays. The prescribed dose was 50 Gy in 25 fractions (2 Gy per fraction, five fractions weekly). Dosimetric constraints followed the guidelines of the Radiation Therapy Oncology Group (RTOG). Doses to all organs at risk (OARs), including bilateral femoral heads, bone marrow, bladder, rectum, and small intestine, were within acceptable tolerances. In November 2021, the patient developed abdominal pain. PET-CT imaging revealed tumor metastasis to the lung, pancreas, bone, and abdominal lymph nodes. The patient subsequently received six cycles of chemotherapy with bevacizumab, irinotecan, and carboplatin, and the tumor achieved partial remission. The patient died of disease 26 months after the initial diagnosis of cervical cancer in November 2022.

### Timeline

See **Table 2**.

**Table 2:** Clinical timeline of a 48-year-old patient with cervical mixed SCNEC and adenocarcinoma accompanied by widespread genital tract dissemination.

Date	Key findings and interventions
Feb 19, 2020	HPV18 positive, TCT showed no intraepithelial lesion or malignancy (NILM)
Aug 28, 2020	Biopsy: Malignancy
Sep 07, 2020	Pathological diagnosis: High-grade neuroendocrine carcinoma with focal glandular differentiation
Sep 15, 2020	Chest CT: No abnormalities in bilateral lungs and mediastinum
Sep 16, 2020	Pelvic MRI: Cervical mass, suspicious for cervical carcinoma. Endoscopy: No abnormalities of esophagus, stomach and colorectum
Sep 21, 2020	Tumor markers: CA-125 153.8 U/mL, CA19-9 68.43 U/mL, ferritin 493.02 ng/mL
Sep 23, 2020	Surgery: Total hysterectomy + bilateral salpingo-oophorectomy + pelvic lymphadenectomy + omentectomy
Oct 05, 2020	Chemotherapy: Etoposide + Cisplatin, 1 cycle
Oct 09, 2020	Pathological diagnosis: cervical mixed SCNEC and adenocarcinoma accompanied by widespread genital tract dissemination, FIGO IB2
Oct 28, 2020	Radiotherapy: vaginal stump and pelvic lymph node drainage region
Oct 29, 2020	Chemotherapy: Etoposide + Cisplatin, 1 cycle
Nov 25, 2020	Chemotherapy: Etoposide + Cisplatin, 1 cycle
Nov 13, 2021	PET-CT: Metastases in lungs, pancreas, bones and abdominal lymph nodes
Nov 15, 2021	Chemotherapy: Bevacizumab + Irinotecan + Carboplatin, 6 cycles
Nov 2022	Patient death

## Discussion

MiNENs represents a heterogeneous group of biphasic differentiated epithelial malignant tumors, most of which are highly aggressive. Primary cervical MiNENs are extremely rare in clinical practice. Fewer than 30 relevant cases have been reported in the literature since 1996 (7–28). In this study, we summarized the clinicopathological features of previously reported cases and the present patient (**Table 1**). The patients ranged in age from 31 to 77 years, with a median age of 48 years. The main clinical manifestations include irregular vaginal bleeding and abnormal vaginal discharge. Gynecological examination revealed cervical erosion accompanied by masses or polypoid lesions, thickened and indurated cervical canal that bled easily on contact. Colposcopy showed obviously thickened acetowhite epithelium and reticular proliferative vascular networks in the lesion area, with mustard-yellow staining after iodine application. Most results of cervical cytological examinations were abnormal. Imaging findings were non-specific, making differentiation from pure-component cervical carcinoma difficult.

Among the 27 reported patients with cervical MiNENs, HPV infection was identified in 19 cases, including 14 cases of HPV18 infection and 6 cases of HPV16 infection, and 5 patients were coinfected with both subtypes. The neuroendocrine tumor components consisted of 13 cases of SCNEC, 13 cases of LCNEC and 1 case of grade 1 neuroendocrine tumor (28). Among the non-neuroendocrine tumor components, there were 25 cases of adenocarcinoma and 2 cases of squamous cell carcinoma. Adenocarcinomas were predominantly HPV-associated adenocarcinomas, including usual-type adenocarcinoma and mucinous adenocarcinoma. Furthermore, 2 gastric-type adenocarcinomas, 1 mesonephric-type adenocarcinoma and 1 adenoid cystic carcinoma were identified, all of which were HPV-unrelated (16, 18, 24, 27).

In terms of pathological diagnosis, MiNENs can be readily diagnosed by combining tumor morphology with immunohistochemistry. The challenge lies in differentiating it from metastatic neuroendocrine carcinoma. In this patient, preoperative chest CT and endoscopic examinations revealed no detectable tumors in the lungs or digestive tract. The tumor cells were negative for CDX-2. Although a small subset of tumor cells exhibited weak TTF-1 immunoreactivity, the staining extent and intensity were inferior to those typically observed in pulmonary small cell carcinoma. Together with the preoperative ancillary examinations, these findings essentially excluded metastatic carcinoma of pulmonary or gastrointestinal origin. Moreover, the neuroendocrine component of the tumor diffusely expressed p16, and PCR confirmed the presence of HPV-18 DNA in the tumor, suggesting that tumorigenesis may be associated with HPV infection (29–31). Collectively, these findings strongly support a primary cervical origin. Of note, focal TTF-1 immunoreactivity, as observed in the present case, does not automatically imply a pulmonary primary, as it has been repeatedly described in primary cervical neuroendocrine carcinomas, but can serve as a reference marker supporting neuroendocrine differentiation (11, 22).

Another diagnostic challenge in this case was the simultaneous detection of adenocarcinoma in the endometrium, fallopian tubes and ovaries besides cervical MiNENs. The lesions in these sites presented features of carcinoma *in situ* (endometrium and fallopian tubes) or borderline tumor (ovary), which easily led to misdiagnosis as primary tumors of the corresponding organs. Upon careful morphological observation, we noted that the tumors of the endometrium and fallopian tubes grew contiguously with the adjacent normal mucosa but had distinct boundaries. Tumor cells appeared to spread along the mucosal layer and replace normal epithelial cells. Although the ovarian lesion exhibited relatively bland morphology, the extravasated mucus in the stroma and desmoplastic reaction of the surrounding stroma indicated that it was not a primary borderline ovarian tumor, but invasive destruction caused by metastatic tumor cells. In addition, the presence of mucous cells within tumor glands, as well as mitotic figures and apoptotic bodies at the glandular luminal margins, were consistent with cervical adenocarcinoma. Immunohistochemically, the tumor cells were negative for ER, PR, vimentin and WT-1, and displayed wild-type p53 expression, further ruling out carcinoma *in situ* arising from the endometrium and fallopian tubes. Diffuse and strong p16 staining combined with the detection of HPV-18 DNA suggested that the adenocarcinomas at these sites were associated with HPV infection and represented spread of cervical adenocarcinoma along the genital tract. Ramalingam et al. proposed that mucosal involvement of the lower uterine segment serves as the histological basis for the spread of early invasive carcinoma or even carcinoma *in situ* of cervical origin to the ovaries (12). Synchronous tumors occurring at multiple sites of the genital tract are most likely attributable to retrograde transmission of HPV within the genital tract (32, 33). Although synchronous HPV-associated Müllerian tract tumors remain a theoretical consideration, this possibility was deemed highly unlikely in the present case, given the unifocal cervical epicenter, the contiguous pattern of involvement, and the consistent HPV18 profile across all anatomic sites.

Cervical mixed neuroendocrine and non-neuroendocrine neoplasms (MiNENs) most commonly consist of neuroendocrine carcinoma combined with HPV-associated adenocarcinoma. These tumors exhibit biological behaviors similar to neuroendocrine carcinoma (NEC) and have a worse prognosis than ordinary carcinomas. As observed in this case, the tumor emboli identified were composed of small cell neuroendocrine carcinoma, and the patient developed multiple systemic metastases 14 months after surgery. Pathologists performed adequate sampling and serial sectioning of the endometrium, bilateral fallopian tubes and ovaries. Repeated HE staining and immunohistochemical staining failed to detect any SCNEC component in these tissues. Adenocarcinoma and small cell neuroendocrine carcinoma in this case exhibit distinctly different patterns of local spread. This difference may be attributed to distinct expression profiles of adhesion molecules (such as E-cadherin) and chemokine receptors between the two components, leading to distinct dissemination preferences—the adenocarcinoma component tending toward infiltration along mucosal surfaces, while the small cell component favors hematogenous spread. In addition, local microenvironmental factors, including immune surveillance and stromal components, may exert differential effects on the two components. Nevertheless, the above mechanisms remain speculative and warrant further investigation.

Radical surgery combined with chemoradiotherapy is the first-line comprehensive treatment for neuroendocrine carcinoma, and cisplatin plus etoposide is the widely adopted chemotherapy regimen. Even so, the overall prognosis of patients remains poor (34). Treatment options for recurrent tumors are quite limited. Delta-like ligand 3 (DLL3) is expected to emerge as a new therapeutic target, paving a new avenue for the follow-up treatment of this disease (35–37).

The pathogenesis and molecular characteristics of MiNENs remain unclear. With the advancement of sequencing technologies, however, available data suggests that the two components of MiNENs are clonally related and originate from a common progenitor cell, such as pluripotent stem cells in the basal layer of the cervical epithelium. These cells differentiate into two distinct tumor lineages, and the two components share identical molecular alterations, which accounts for the ill-defined morphological boundaries between them (18, 28, 38).

## Conclusion

Herein, we report a case of primary cervical MiNENs associated with HPV18 infection, which was composed of HPV-associated adenocarcinoma and SCNEC. Integrating tumor morphological, immunophenotype and multi-site HPV DNA detection results, these findings strongly support that bilateral ovarian adenocarcinoma arise from the metastatic extension of cervical adenocarcinoma via the endometrium and fallopian tubes. Persistent infection with high-risk HPV and its retrograde dissemination along the genital tract represent important factors driving distant metastasis of this tumor. Nevertheless, we acknowledge that without molecular clonality assays, the dissemination pathway, although strongly supported by the current data, cannot be regarded as definitively proven. We will continue to enroll similar cases, and future studies incorporating clonality analysis would be valuable to further corroborate this interpretation.

## Data Availability

The original contributions presented in the study are included in the article/supplementary material. Further inquiries can be directed to the corresponding author.
